# Click-on fluorescence detectors: using robotic surgical instruments to characterize molecular tissue aspects

**DOI:** 10.1007/s11701-022-01382-0

**Published:** 2022-04-09

**Authors:** Matthias N. van Oosterom, Sven I. van Leeuwen, Elio Mazzone, Paolo Dell’Oglio, Tessa Buckle, Florian van Beurden, Michael Boonekamp, Huybert van de Stadt, Kevin Bauwens, Hervé Simon, Pim J. van Leeuwen, Henk G. van der Poel, Fijs W. B. van Leeuwen

**Affiliations:** 1 Interventional Molecular Imaging Laboratory, Department of Radiology, Leiden University Medical Center, Leiden, The Netherlands; 2 Department of Urology, Netherlands Cancer Institute-Antoni van Leeuwenhoek Hospital, Amsterdam, The Netherlands; 3 Department of Urology and Division of Experimental Oncology, Urological Research Institute, IRCCS San Raffaele Scientific Institute, Milan, Italy; 4 ORSI Academy, Melle, Belgium; 5 Department of Urology, ASST Grande Ospedale Metropolitano Niguarda, Milan, Italy; 6 Design & Prototyping, Department of Medical Technology, Leiden University Medical Center, Leiden, The Netherlands; 7 Eurorad, Eckbolsheim, France

**Keywords:** Robotic surgery, Fluorescence guided surgery, Image-guided surgery, Biosensing, Near-infrared, Steerable surgical instruments

## Abstract

Fluorescence imaging is increasingly being implemented in surgery. One of the drawbacks of its application is the need to switch back-and-forth between fluorescence- and white-light-imaging settings and not being able to dissect safely under fluorescence guidance. The aim of this study was to engineer ‘click-on’ fluorescence detectors that transform standard robotic instruments into molecular sensing devices that enable the surgeon to detect near-infrared (NIR) fluorescence in a white-light setting. This NIR-fluorescence detector setup was engineered to be press-fitted onto standard forceps instruments of the da Vinci robot. Following system characterization in a phantom setting (i.e., spectral properties, sensitivity and tissue signal attenuation), the performance with regard to different clinical indocyanine green (ICG) indications (e.g., angiography and lymphatic mapping) was determined via robotic surgery in pigs. To evaluate in-human applicability, the setup was also used for ICG-containing lymph node specimens from robotic prostate cancer surgery. The resulting Click-On device allowed for NIR ICG signal identification down to a concentration of 4.77 × 10^–6^ mg/ml. The fully assembled system could be introduced through the trocar and grasping, and movement abilities of the instrument were preserved. During surgery, the system allowed for the identification of blood vessels and assessment of vascularization (i.e., bowel, bladder and kidney), as well as localization of pelvic lymph nodes. During human specimen evaluation, it was able to distinguish sentinel from non-sentinel lymph nodes. With this introduction of a NIR-fluorescence Click-On sensing detector, a next step is made towards using surgical instruments in the characterization of molecular tissue aspects.

**Supplementary Information:**

The online version contains supplementary material available at 10.1007/s11701-022-01382-0.

## Introduction

One of the key features through which surgery can be improved is intraoperative target identification. In the last decade we have seen a rise of fluorescence guidance as a means to facilitate the decision-making process during (laparoscopic) surgery. In particular, angiography and lymphatic mapping with the near-infrared (NIR) fluorescent dye indocyanine green (ICG) are now widely used [1]. The clinical demand for fluorescence-guided surgery coupled with the competition between industries that supply surgical equipment has given rise to a wealth of fluorescence detection modalities. Most engineering efforts focus on integrating fluorescence guidance in routine surgical workflows. For example, the first clinical fluorescence-guided surgery studies with the da Vinci robot still relied on the introduction of a second laparoscope through the assistant port to enable fluorescence imaging [2]. However, current da Vinci robotic platforms are now equipped with a Firefly fluorescence laparoscope designed to support white light imaging as well as the detection of ICG [3]. As such, the full da Vinci install base can benefit from ICG fluorescence guidance. A feature that has allowed surgeons to explore a variety of fluorescence-guided surgery concepts for different surgical indications; e.g., lymph node (LN) dissection [4, 5], partial nephrectomy [6, 7], anastomosis of the bowel [8], visualization of the ureters [1] and biliary tree identification [9, 10].

Where ICG has been one of the few fluorescent tracers currently used in routine care, this does not mean that the technology of fluorescence-guided surgery is no longer evolving. In fact, the field is actively being extended through the introduction of a great variety of fluorescent tracers [11, 12]. Furthermore, engineering efforts in fluorescence-guided surgery try to explore concepts such as reduced dosing [13, 14], lifetime-imaging [15], use of different and/or complementing fluorescent emissions [12], optoacoustic imaging [16], and fluorescence tomography [17]. One large constraint in all these fluorescence guidance concepts is that their implementation requires a surgeon to sequentially apply fluorescence and white light imaging. Surgeons are trained to resect under white light conditions and these conditions provide the best way to assess the surgical field (e.g., bleedings). As such, switching between different imaging modes can cause pauses that are detrimental to the surgical workflow.

The aim of this project was to introduce a ‘molecular sensing’ technology that can measure the fluorescent content present in all tissues grasped during robotic surgery. This information source should be independent of the surgical light settings used (i.e., white light or fluorescence imaging) and the type of laparoscope used. Building on an earlier ‘click-on’ detector design [18] and optical fluorescence tracing concepts [17, 19], we engineered a Click-On fluorescence detection system that can be attached to the widely applied ProGrasp instrument, providing both acoustic and numerical feedback upon the presence of ICG in the grasped tissues. This technology was evaluated in a phantom setting, during porcine surgery, and on surgical specimens (i.e., sentinel lymph nodes; SNs) from prostate cancer surgery.

## Methods

### Engineering of the Click-On NIR fluorescence sensing device and system characterization

**Fig. 1:**
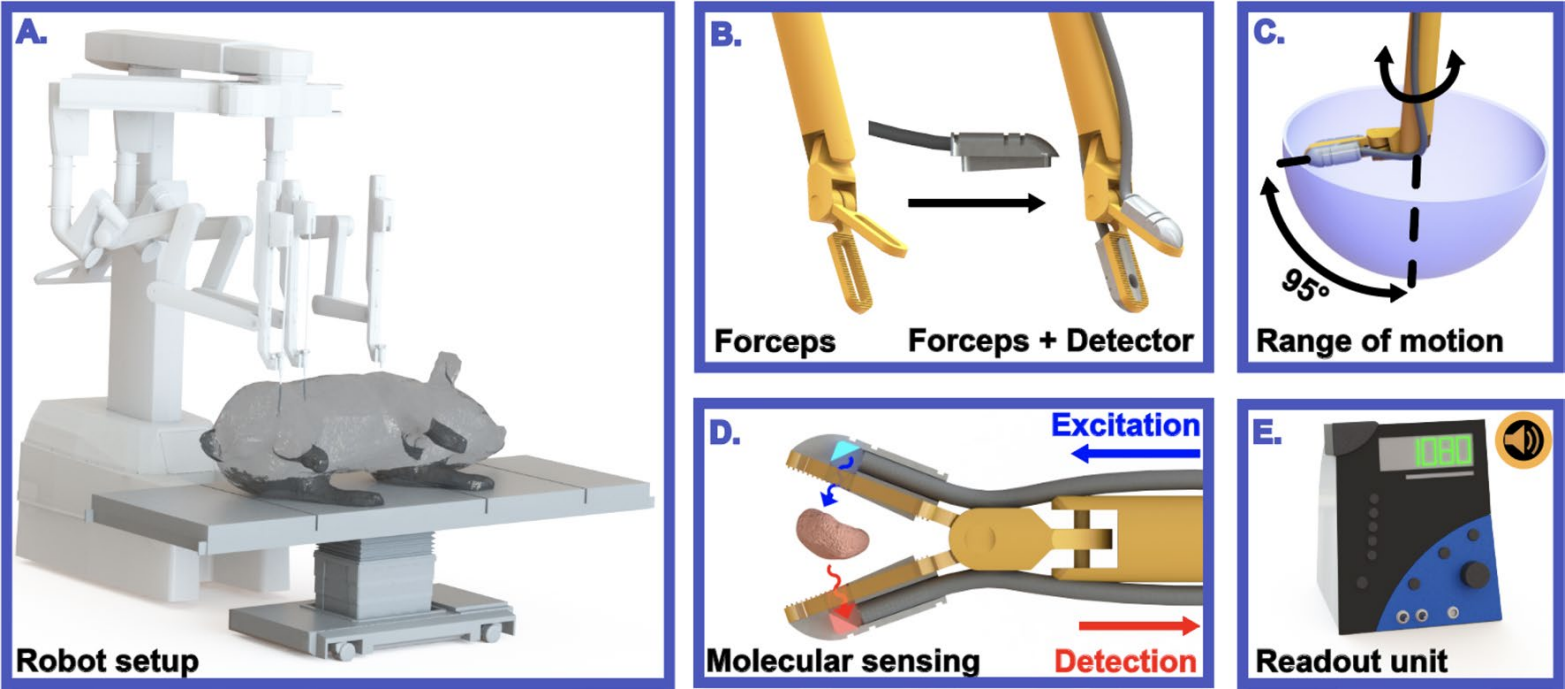
Schematic overview of the NIR fluorescence Click-On setup. **A**–**B** The modules are clicked onto a standard ProGrasp Forceps of the Da Vinci surgical robot. **C** The range of motion of the steerable instrument is not limited by the Click-On modules. **D** Upon illumination with the excitation light (in blue), the instrument detects the fluorescent tracer (in red) between the jaws of the forceps. **E** Upon detection of NIR fluorescence, the read-out unit provides both a numerical (i.e., counts/s) and audible feedback

A schematic overview of the Click-On setup is shown in Fig. 1. It consists of excitation and detection modules, designed to press-fit on to the jaws of a ProGrasp Forceps robotic instrument (Fig. 1D). The prototype was built using an OptoNuclear read-out console (Eurorad SA) as basis [17, 19]. Both excitation and emission modules consist of a computer numerical control (CNC)-milled aluminum housing, designed with computer-aided design (CAD) software (SolidWorks, Dassault Systèmes SA), a CNC-milled stainless-steel mirror and acrylic optical fiber (Edmund Optics Inc., Barrington, NJ, USA). Two acrylic optical fibers transport fluorescence excitation and emission and each have a 1 mm diameter and 0.51 numerical aperture. The excitation side is coupled to a 785 nm laser for fluorescence excitation, while the emission side is coupled to an optically filtered (i.e., > 810 nm) infrared-extended photomultiplier tube (H10721-20, Hamamatsu Photonics kk.) for fluorescence emission collection. Sample time for detection was chosen as 0.5 s. Fluorescence detection was depicted as both a numerical (i.e., counts/s) and audible read-out (Fig. 1E).

### Excitation properties

To characterize the illumination properties of the system with respect to the absorption and emission spectra of ICG, a Horiba Jobin Yvon VS140 linear array fiber spectrometer (Horiba Ltd., Kyoto, Japan) was used with a 1 m optical fiber patch cable (M15L01, Thorlabs Inc., Newton, NJ, USA). To investigate the actual excitation power within the jaws of the Click-On system, a Thorlabs PM16-121 power meter (Thorlabs Inc.) was used with National instruments VISA software set at 785 nm.

### Detection properties

To characterize the sensitivity of NIR fluorescence detection of the resulting Click-On setup, glass capillaries were taken (inner diameter 1.15 mm, outer diameter 1.55 mm; Micro Haematocrit tubes, Brand GmbH) and filled with an ICG dilution series ranging from 5.0 mg/mL to 9.3 ng/mL, dissolved in a human serum albumin concentration (HSA; 200 g/L) with dilution steps of 1:1, similar as used before [20]. All individual capillaries were grasped with the forceps setup and the resulting count rates were recorded. The measurements were acquired in triplicate. To demonstrate the effect that tissue has on these measurements, the whole process was also repeated with porcine fatty tissue covering the capillaries on all sides with a layer of approximately 1 mm, which would result in a rough tissue volume of 50 mm^3^.

To compare sensitivity with respect to the Firefly fluorescence camera systems of the da Vinci Si and Xi robotic systems, the same ICG dilution series was measured with both Firefly Si and Xi systems [20]. Since the cameras are applied in an observative fashion, rather than a grasping fashion, the ICG dilutions were placed in a black well-plate (96-wells, Cellstar; Greiner Bio-One GmbH, Frickenhausen, Germany; with each well containing 50 μL of solution).

Opening and closing of the forceps jaws changes both the illumination profile on the targeted tissue (i.e., excitation light becomes more or less focused), as well as the captured emission profile of the targeted tissue (i.e., more or less fluorescent emission is captured). Therefore, measurements were taken to evaluate how sensitivity of ICG fluorescence detection changes at different opening angles of the forceps jaws. To this end, the fluorescent counts/s were measured with the setup using various jaw angles and a capillary filled with an ICG concentration of 7.63 × 10^−5^ mg/mL (prepared as described above). To fix the opening angles of the jaws precisely and reproducibly, a small holder was designed with CAD software (Dassault Systèmes SA) and 3D printed (acrylonitrile butadiene styrene plastics; Dimension Elite 3D printer, Stratasys Ltd., Eden Prairie, MN, USA), to fixate the capillary and the jaws at defined angles (ranging from 10 to 35 degrees). The whole experiment was performed four times.

### In vivo evaluation in porcine models

Functioning of the NIR Click-On setup during surgery was investigated in five living porcine models (weight per animal approximately 40 kg), with no specific inclusion or exclusion criteria. Using both the da Vinci Si and Xi robotic platforms, two different surgical applications were investigated: (1) tissue vascularization detection, and (2) LN detection. In the first, ICG was injected intravenously (1.5 mL, 2.5 mg/mL solution in sterile water) roughly 1 h prior to surgery. For the latter, ICG (~ 0.1 mL, 2.5 mg/mL solution in sterile water) was injected intraoperatively using a needle attached to a syringe via a flexible tubing system, as previously described [21]. After insertion of the needle through the trocar, the surgeon placed several tracer deposits at different anatomical locations to induce drainage towards LNs. To cross-validate functioning of the Click-On setup intraoperatively, the Firefly robotic laparoscope was used to confirm the presence of ICG via fluorescence imaging.

Tissue strongly attenuates light, mostly due to absorption and scatter [22]. As such fluorescence signal intensities are generally considered qualitative measures. In fact, in vivo quantification does not occur during routine clinical use of ICG. To still investigate if the quantification of fluorescence signals measured with the Click-On system would be of added benefit, a rough comparison was made between: (1) the theoretical ICG concentration expected in the tissue of interest based on injected dose and tracer pharmacokinetics, and (2) the ICG concentration that would be estimated based on the numerical read-out as provided by the Click-On system.

All animal experiments were performed under approval by the ethical board of the University of Ghent and were performed in accordance with the Experiments on Animals Act (Wod, 2014), the applicable legislation in Belgium and in accordance with the European guidelines (EU directive no. 2010/63/EU) regarding the protection of animals used for scientific purposes. No animals had to be excluded from the evaluation. All animal data were reported in accordance with the ARRIVE guidelines (Animals in Research: Reporting In Vivo Experiments) [23].

### Ex vivo evaluation with prostate cancer samples

To evaluate the NIR Click-On setup with clinically relevant doses in human tissue, surgical LN specimens of three prostate cancer patients were evaluated ex vivo with the da Vinci Si robotic platform. These patients underwent a robot-assisted laparoscopic SN biopsy procedure, extended pelvic LN dissection and radical prostatectomy. To guide intraoperative dissection of the SNs, either the hybrid tracer ICG-^99m^Tc-nanocolloid (2 patients) or ^99m^Tc-nanocolloid (1 patient) was injected intra-prostatically using four deposits, in the morning before surgery, similar as described previously [24]. To allow for fluorescence guidance in the latter group, ICG was injected intra-prostatically at the start of surgery. Using both lymphoscintigraphy and single-photon emission computed tomography/x-ray computed tomography (SPECT/CT) imaging, the number and location of prostate draining SNs were mapped before surgery. After surgical removal in the afternoon, the Click-On NIR detectors were applied ex vivo to evaluate if it could discriminate between the SNs that contained tracer uptake and the LNs which did not. Besides using the Firefly fluorescence laparoscope to confirm the presence of fluorescence, in this setting using a bimodal tracer, radioguidance (NeoProbe, Devicor Medical Products) also allowed for cross-validation. For ex vivo confirmation of fluorescence imaging, an open surgery fluorescence camera was used (FIS-00, Hamamatsu Photonics K.K., Japan). All activities were in accordance with the ethical approval of our institution and the Helsinki Declaration.

## Results

### Engineering of the Click-On NIR fluorescence sensing device and system characterization

The Click-On fluorescence system was designed to: (1) obtain sufficient sensitivity (i.e., emit enough light for excitation and collect enough of the emission light), (2) allow for the fully mounted instrument to be inserted through a 12 mm trocar, (3) not restrict the 6-degrees of rotational freedom of the ProGrasp forceps [25] maintaining a rough range of movement from − 95° to + 95° around the nominal axis of the instrument, and (4) preserve the normal grasping functions of the ProGrasp forceps (Fig. 1).

### Excitation properties

**Fig. 2:**
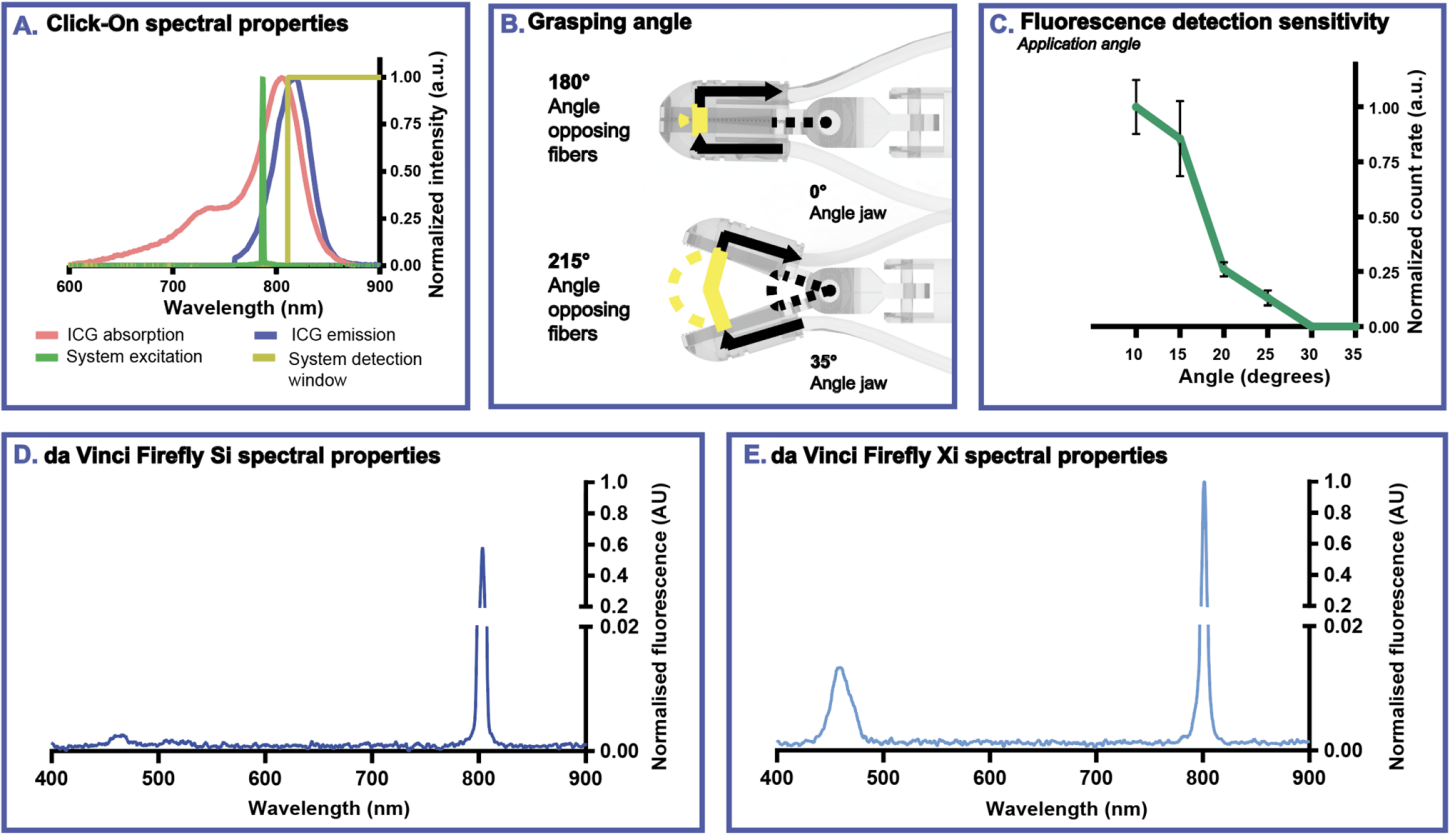
Characterization of the Click-On NIR fluorescence device and Firefly Si and Xi camera systems. **A** An overview of the relevant spectral properties of the Click-On system (i.e., excitation in green and detection window in yellow) in relation to the absorption (in red) and emission spectra (in blue) of ICG. **B** Graphical representation of the forceps jaws in a closed position (top) and an open position (bottom). **C** Influence of the forceps jaw angle on fluorescence detection sensitivity (without tissue). **D** Excitation spectrum of the Firefly Si system. **E** Excitation spectrum of the Firefly Xi system

An overview of the spectral properties of the NIR Click-On system, the Firefly Si in fluorescence mode and Firefly Xi in fluorescence mode are shown in Fig. 2A, D and E, respectively. The custom-made mirrors (placed over an angle of 45°) were sufficient to angle the excitation light and fluorescent emission in the jaws of the instrument, out of and into the fibers respectively. Measuring the light intensity with the power meter revealed 1.8 mW within the grasping surface of the forceps jaws (~ 5 mm^2^). By using a press fit (i.e., the Click-On parts were manufactured 0.05 mm larger than the inner cavity of the grasper), we were able to explore free space in the jaws, thus preserving the geometry (i.e., the grasping surface) of the grasper. In addition to the audible feedback, the Click-On detection system provides a count rate proportional to the amount of fluorescence detected, both reflecting the number of counts per second.

### Detection properties

Evaluation in the phantom setup showed that, without a fluorescent source present, the Click-On modules did not record signal (counts/s); neither in open (opposing fibers at > 215°) or closed position (opposing fibers at 180°; Fig. 2B). As is shown in Fig. 2C, the opening angle of the forceps jaws had a large influence on the ICG detection sensitivity: the smaller the opening angle the higher the detection sensitivity. Beyond an opening angle of about 30°, the forceps was no longer able to accurately detect ICG fluorescence in this phantom setting.

**Fig. 3:**
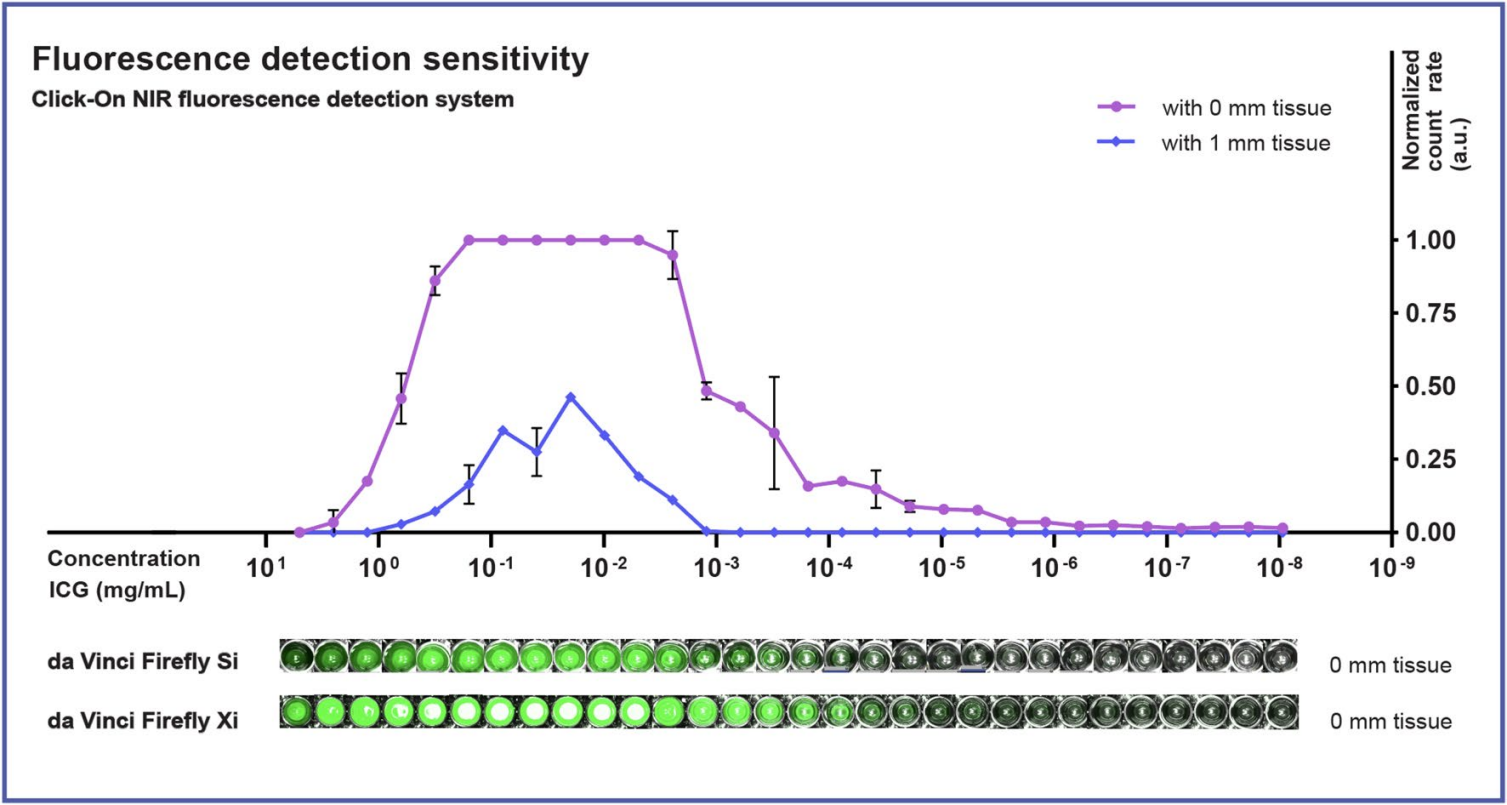
Fluorescence detection sensitivity of the Click-On NIR fluorescence device and Firefly Si and Xi camera systems

An overview of the count rate detected for the ICG dilution series in the capillaries with and without tissue is shown in Fig. 3. Without tissue present, an increasing ICG concentration resulted in an increased detected count rate, up to a concentration where the dye self-quenches, as described previously [20]. Signal saturation was reached for concentrations between 3.13 × 10^–1^ and 2.44 × 10^–3^ mg/mL. The detection limit in this NIR Click-On setup was found to be at an ICG concentration of at least 4.77 × 10^–6^ mg/mL. Repeating the measurements of the dilution series using attenuation by fatty porcine tissue (50 mm^3^) yielded a clear signal attenuation, resulting in a significant drop in signal intensity and detection range (detection limit at 6.10 × 10^–4^ mg/mL), but preserved the same detection trend. Figure 3 also displays the detection sensitivity of both the Firefly Si and Xi camera systems with respect to the Click-On detector, suggesting the latter has a superior sensitivity.

### In vivo evaluation in porcine models

**Fig. 4:**
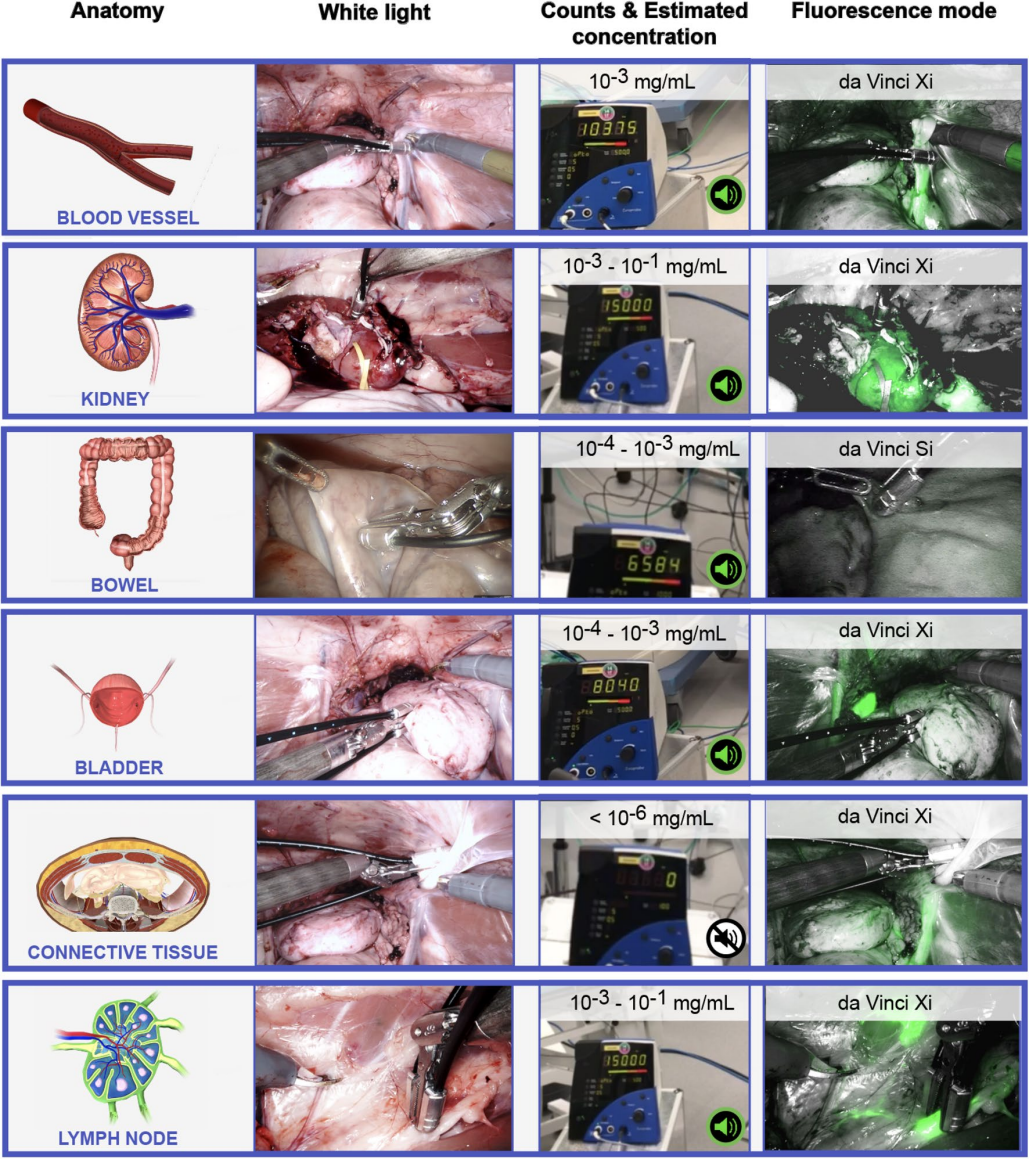
Overview of the in vivo evaluation of the Click-On NIR fluorescence setup, showing examples for different applications. The Click-On modality functions in both white light imaging mode and fluorescence imaging mode, rendering it independent of the laparoscopic camera setting. The Click-On modality provides both an audible and numerical feedback once ICG is detected. The latter is used the roughly estimate the ICG concentration in the tissue grasped

Figure 4 shows an overview of the Click-On system functioning in various laparoscopic applications during robotic surgery in pigs (see also Supplemental Video 1). Hereby, the count rates displayed demonstrate typical examples of the count rates detected. Given the high values obtained in vivo, it is likely that the ICG uptake measured was within a 1 mm tissue depth for all tissues (see Fig. 3 for count rates measured with and without tissue).

In the angiographic experiments, it was possible to identify individual blood vessels or evaluate the vascularization of a specific organ section, such as for the kidney, bowel and bladder (Fig. 4), with Click-On count rates detected up to 15,000 counts/s, at which the system was fully saturated. Based on the intravenously injected amount of ICG (i.e., 3.75 mg) and the typical total blood volume of a pig (i.e., ~ 2.6 L for a pig of 40 kg), it is expected that a well vascularized part of tissue would contain 10^–3^ mg/mL of ICG. Figure 4 displays a count rate 10,375 counts/s measured with the Click-On directly on a blood vessel. Relating this count rate to the values displayed in Fig. 3 would also translate to a concentration range of 10^–3^ mg/mL. Figure 4 shows a count rate of 15,000 counts/s for a vascularized part of the kidney, which would translate to an ICG concentration of 10^–3^–10^–1^ mg/mL. In a similar fashion, the examples from Fig. 4 show a rough estimated ICG concentration of 10^–4^–10^–3^ mg/mL for both bowel and bladder tissue. Background measurements in control tissue (e.g., connective tissue; as observed with the Firefly) helped to confirm the systems specificity, displaying a count range of 0 counts/s (< 10^–6^ mg/mL; Fig. 4). All count rates observed were in line with the fluorescence intensities identified using the Firefly laparoscope (Fig. 4). The Click-On modules provided a successful performance in all light settings and were independent of the type of Firefly laparoscope used, rendering the Click-On modality independent of the camera settings and allowing for fluorescence detection during every part of the procedure.

In the pigs wherein ICG was locally administered (e.g., in abdominal wall or muscle) to allow for lymphatic mapping, the Click-On system was able to successfully localize the tissue draining LNs accumulating ICG. Based on the injected amount of ICG (i.e., 0.25 mg), typical lymphatic drainage (i.e., 1–10% [13]) and typical lymph node volume (i.e., roughly 0.15 mL), it is expected that there would be an ICG concentration of 10^–3^–10^–2^ mg/mL in the draining lymph nodes. Again, relating the count rate displayed in Fig. 4 (15,000 counts/s) to the sensitivity displayed in Fig. 3, yields a matching ICG concentration range (10^–3^–10^–1^ mg/mL). Just as with the angiographic applications, the Click-On NIR fluorescence measurements were possible during both white light and fluorescence imaging settings of the Firefly (Fig. 4).

### Ex vivo evaluation with prostate cancer samples

**Fig. 5:**
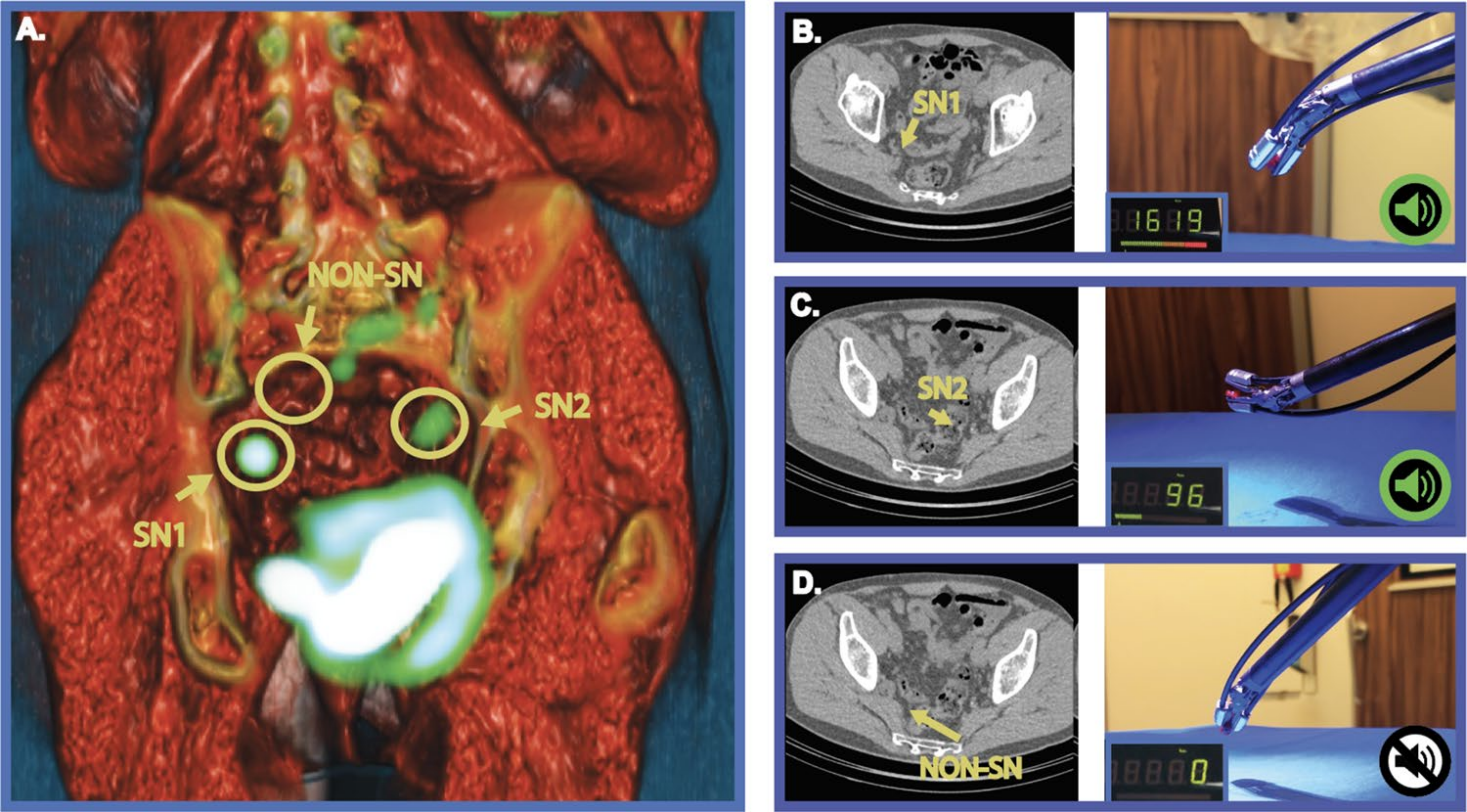
Example of the ex vivo evaluation in prostate cancer SN surgery. **A** A volume rendering of the patient SPECT/CT scan shows an overview of two SN locations, where tracer uptake is visualized in green. To illustrate the location of the non-SN specimen removed during surgery, an annotation of this lymph node was also added to the scan. **B**–**D** The three annotated lymph nodes are shown on the individual CT slice (left) and evaluated with the Click-On fluorescence setup (right). The Click-On modality was able to identify SN specimens from non-SN specimens, providing a non-zero count rate and a sound when the SNs were detected

Based on the preoperative SPECT/CT (Fig. 5A), SN locations were mapped and intraoperatively resected using both fluorescence- and radio-guidance, as described previously [13]. After surgical removal of the prostate cancer related (sentinel) LN specimens, back-table evaluations with the Click-On NIR fluorescence system enabled for a clear separation between LNs containing ICG-^99m^Tc-nanocolloid (count rates up to 4375 counts/s) and those that did not (count range of 0 counts/s; Fig. 5). This was confirmed with the Firefly fluorescence laparoscope, the FIS-00 open surgery fluorescence camera and a handheld gamma probe. During these analyses, the readout of the Click-On NIR fluorescence detectors was not limited by ambient operating room lightening, a light source known to impair fluorescence detection [26].

## Discussion

**Fig. 6:**
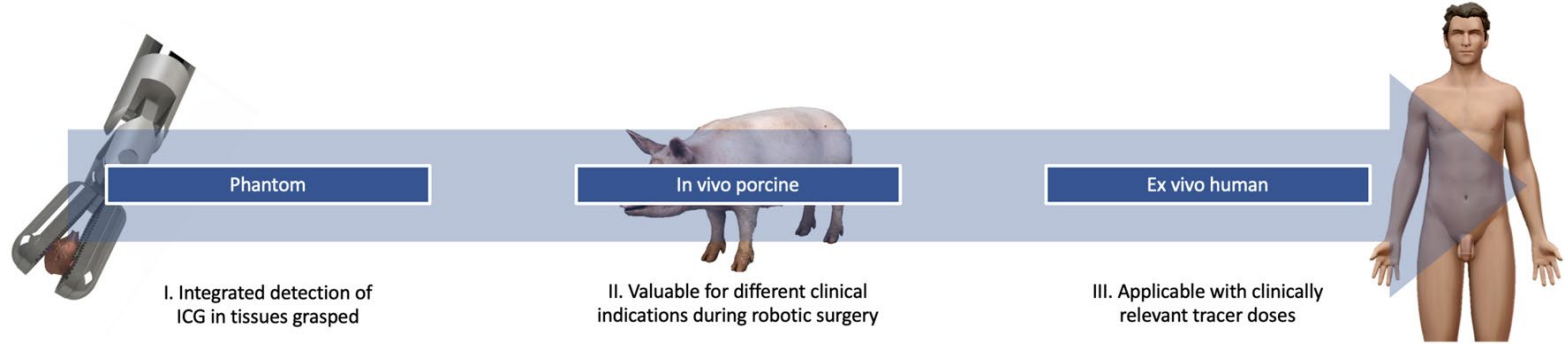
Overview of the Click-On fluorescence detector evaluation, displaying the three main stages: I. phantom, II. in vivo porcine and III. ex vivo human

By creating a Click-On NIR fluorescence detector, we have been able to convert a ProGrasp forceps into a fluorescence sensing device. Providing integrated detection of ICG, the technology proved value during real-life robot-assisted procedures, such as angiography (including e.g., bladder and bowel anastomoses) and ICG lymphatic mapping, even at clinically relevant doses (Fig. 6). In these procedures the instrument provides a numeric and acoustic read-out that complements the images of the fluorescence laparoscope. Important to note is that this type of fluorescence guidance remained useful in the white-light imaging mode, thus allowing the operating surgeon to “sense” the fluorescence signal during every part of the procedure, including the actual dissection. As such, this may also provide a way to realize fluorescence detection on newly emerging clinically approved robotic platforms (e.g., the Hugo RAS system of Medtronic plc and the Versius system of CMR Surgical Ltd) that do not yet have integrated fluorescence cameras.

Through the opto-nuclear technology, fiber based fluorescent sensors have already demonstrated clinical potential in both open and laparoscopic surgery [17, 19, 27, 28]. Alternatively, fiber-based detection has also been clinically explored with for example Raman spectroscopy [29], diffuse reflectance spectroscopy [30], autofluorescence lifetime [31] and confocal microscopy [32]. In the current study we extend on these efforts by integrating optical fibers with the robotic surgery instruments themselves. This approach is in line with the generation of dedicated tethered modalities for (robot-)assisted laparoscopic surgery [33], such as the ‘drop-in’ ultrasound [34], ‘drop-in’ gamma probe [24], ‘drop-in’ beta probe [35], ‘drop-in’ mass spectrometry probe [36] and ‘drop-in’ electrical bio-impedance probe [37]. These modern detection technologies allow the robotic surgeon to autonomously position the detectors with optimized degrees of freedom [25], providing more maneuverability with respect to rigid laparoscopic instruments, including traditional fluorescence laparoscopes. With our Click-On gamma probe efforts we demonstrated that attaching the modality directly to the instrument enhances the surgical dexterity [18]. While our efforts with gamma and NIR-fluorescence detection are based on the ProGrasp instrument, an instrument that is often used as third supporting instrument during the time of dedicated resection, the concept could equally apply to other instruments.

One of the limitations of this proof-of-concept study is the inability to define how the technology would impact patient care. Something that remains a general challenge for the up-and-coming field of fluorescence guided surgery; impact on patient care requires long term outcome studies. That said, one of the largest robotic studies on the value of fluorescence guidance at the moment (*n* = 1680) does indicate the potential benefit that fluorescence could have, indicating that more positive lymph nodes could be retrieved during prostate cancer lymph node surgery [38]. Recently, it was shown that performance improvements realized through a medical device may also be defined via kinematic assessment of surgical instrument movements [18]. In the future, a head-to-head comparison study between the Click-On fluorescence detectors and the current clinical standard could provide quantified insight in to how the technology alters surgical decision making, dexterity and logistics. For routine in-human application, however, the Click-On device will need to comply to the latest European regulations for medical devices (i.e., EU MDR).

Next to the challenges faced to quantify performance improvements, a major challenge is found in the quantification of fluorescence signal intensities. Light is severely attenuated by tissue, as such NIR fluorescence-guided surgery remains a superficial method (i.e., < 1 cm depth penetration) [39]. Some studies, however, suggest that this effect can be partially compensated by compressing the tissue [40]. A concept that we inherently apply in our sensing setup (see Fig. 4), where the fluorescent content is measured when tissue is grasped within the forceps. Therefore, it is likely to expect that a grasping method can detect low fluorescent signatures that are initially not visible using a fluorescence camera. Furthermore, since the fluorescent content of the tissue grasped is measured as counts per second, rough comparison of the fluorescent tracer uptake is possible between different tissues. Interestingly, comparing the theoretical and in vivo count-derivatized ICG concentrations (Figs. 3 and 4) suggested similar ICG concentrations. However, since tissue composition and depth of signal are uncontrollable during surgery, so is the attenuating effect that tissue has on the fluorescent signal intensities. Consequently, we consider real-time quantification of the fluorescence signal intensities in vivo currently too unreliable.

In the current study, we used Click-On detectors on the ProGrasp instrument already in routine clinical use. To avoid additional tissue damage, we did not modify the grasping function of the instruments themselves. That said, caution should still be taken as different tissue types have a different pressure resistance [41]. Hence, random grasping of tissue should be avoided. An argument to use the technology in conjunction with a fluorescence laparoscope.

By engineering the NIR Click-On detectors, we provided a first step towards the generation of fluorescence sensing surgical instruments. Future refinement of this concept could include multispectral (also called ‘multi-wavelength’ or ‘multi-color’) fluorescence detection [12]. This would allow the instrument to “sense” complementary fluorescent emissions (e.g., multiple tracers) during a single surgical procedure [20], e.g., detection of both the primary tumor and tumor draining lymphatics with two individual fluorescent tracers [42].

## Conclusion

The present study successfully introduces the concept of a Click-On fluorescence-based sensing modality for robotic surgical instruments, turning regular forceps into a molecular sensing device. As such the modality was capable of detecting ICG in tissue during every part of the surgery, independent of the fluorescence laparoscope type and settings. This is a step towards biosensing applications, where the surgical instruments themselves can be used to characterize the molecular aspects of tissue.

## Supplementary Information

Below is the link to the electronic supplementary material.

Supplementary file1 (MP4 130402 KB)

## Data Availability

If not already present in the manuscript, all remaining datasets used during the current study are available from the corresponding author on reasonable request.
